# Evolution of Potential Distribution Areas and Cultivation Zones of *Morchella esculenta* (L.) Pers. Under Climate Warming: Application of Ensemble Models and Production Dynamics Models

**DOI:** 10.3390/jof11070475

**Published:** 2025-06-22

**Authors:** Yi Huang, Guanghua Zhao, Jingtian Yang, Liyong Yang, Yang Yang, Wuzhi Jiaba, Zixi Shama, Jian Yang

**Affiliations:** 1 Sichuan Provincial Forest and Grassland Key Laboratory of Alpine Grassland Conservation and Utilization of Tibetan Plateau, College of Grassland Resources, Southwest Minzu University, Chengdu 610041, China; 110055@mtc.edu.cn (Y.H.); smuyangyang@utibet.edu.cn (Y.Y.); 2 Key Laboratory of Biodiversity and Environment on the Qinghai-Tibetan Plateau, Ministry of Education, School of Ecology and Environment, Tibet University, Lhasa 850001, China; 3 Ecological Security and Protection Key Laboratory of Sichuan Province, Mianyang Normal University, Mianyang 621000, China; yjtdc@mtc.edu.cn (J.Y.); 2208570131@stu.mtc.edu.cn (L.Y.); jiabawuzhi@mnu.cn (W.J.); shamazixi@mnu.cn (Z.S.); 4 School of Life Science, South China Normal University, Guangzhou 510631, China; 2024010259@m.scnu.edu.cn

**Keywords:** upper Dadu River–Minjiang River region, *Morchella esculenta* (L.) Pers., climate change, potential distribution, cultivation zones

## Abstract

Under global climate change, sustainable management of plant resources in alpine canyon regions faces severe challenges. *M. esculenta*, highly valued for its edible and medicinal properties, is widely harvested for consumption by residents in the upper Dadu River–Minjiang River region. This study employs ensemble models to simulate the potential distribution of *M. esculenta* in this region, predicting the impacts of future climate change on its distribution, centroid migration of suitable habitats, and niche dynamics. Additionally, a production dynamics model integrating ecological suitability and nutritional components was developed to delineate current and future potential cultivation zones for *M. esculenta*. The results indicate that current high-suitability areas and core cultivation zones of *M. esculenta* are predominantly distributed in a patchy and fragmented pattern. The high-suitability habitats in the upper Dadu River–Minjiang River region have three distribution centers: the largest spans southern Danba County, southern Jinchuan County, and northeastern Kangding City, while the other two are located in northeastern Li County, southwestern Aba County, and northwestern Ma’erkang City, with sporadic distributions in Heishui County, Maoxian County, and Wenchuan County. First-level cultivation areas are primarily concentrated in Kangding City, Danba County, Ma’erkang City, Li County, and surrounding regions. Under climate change, low-suitability areas and third-level cultivation zones for *M. esculenta* in the region have increased significantly, while high- and medium-suitability areas, along with first- and second-level cultivation zones, have decreased notably. Concurrently, suitable habitats and cultivation zones exhibit a migration trend toward higher northern latitudes. The most pronounced changes in suitable areas and cultivation zones, as well as the largest niche migration, occur under the high-emission climate scenario. This study facilitates the formulation of suitability-based management strategies for *M. esculenta* in the upper Dadu River–Minjiang River region and provides a scientific reference for the sustainable utilization of mountain plant resources under climate change.

## 1. Introduction

*Morchella esculenta* (L.) Pers., a precious edible and medicinal fungus belonging to the Ascomycota phylum, derives its name from the honeycomb-like appearance of its cap [1]. It is rich in nutrients such as proteins, carbohydrates, and fats, as well as non-nutritive bioactive components, including dietary fiber, enzymes, and organic acids, exhibiting antioxidant, anti-proliferative, and immunomodulatory properties [2,3,4,5,6]. In recent years, the cultivation of *M. esculenta* has expanded rapidly due to its high economic value, earning it the moniker “the gold of edible fungi” [1]. While its fruiting bodies are challenging to cultivate due to environmental sensitivities, mycelial fermentation offers a viable alternative for producing bioactive compounds, characterized by faster growth rates and shorter fermentation cycles [7,8]. However, existing research has primarily focused on nutritional analysis, medicinal applications, and cultivation techniques [4,5,6,9,10], leaving significant gaps in the understanding of its niche dynamics and habitat responses to climate change. This knowledge deficit is particularly pronounced in alpine ecosystems (mountainous canyon ecosystems), which are highly sensitive to climate fluctuations, where the distribution patterns and sustainable utilization potential of *M. esculenta* remain understudied.

The upper Dadu River–Minjiang River region exhibits a distinct northwest–southeast topographic gradient, transitioning northward to the Qinghai-Tibet Plateau and southward to the Sichuan Basin. As an important ecological security barrier and headwater protection area in the upper Yangtze River, this region features a unique three-dimensional landform integrating plateau, mountain, and valley systems. Its complete vertical vegetation zones include four defined altitude belts: arid valley shrublands (2000–2800 m asl), montane coniferous–broadleaved mixed forests (2800–3600 m asl), subalpine dark coniferous forests (3600–4200 m asl), and alpine meadow–talus zones (>4200 m asl). This remarkable environmental heterogeneity—characterized by huge altitude gradients and complex microclimate differentiation—has made the watershed a globally recognized biodiversity hotspot [11]. This region exhibits a dual ecological and socioeconomic profile: a “mountainous canyon barrier” that fosters unique biodiversity and a “resource-constrained livelihood” system reliant on traditional subsistence practices [12]. Field surveys confirm that *M. esculenta* represents a highly valued dietary resource among communities in the upper Dadu River–Minjiang River region. Market analyses document premium dried specimens commanding prices up to CNY 300 per 500 g and fresh specimens at CNY 120 per 500 g, underscoring their substantial economic importance within local subsistence and commercial systems. Recent climate observations reveal significant shifts in hydrothermal regimes, with warming rates exceeding global averages, intensified precipitation variability, and increased frequency of drought events [12,13,14]. These changes have already impacted the phenology and fruiting yields of *M. esculenta* [8]. Therefore, assessing its habitat suitability and cultivation potential in this region is critical for both biodiversity conservation and sustainable development of mountain communities.

At the macro level, the climate is a primary determinant of species distribution, and adaptive responses to climate variability shape ecological diversity [15,16,17]. Climate change disrupts species growth and population dynamics, driving range shifts and distributional changes [17,18]. Global climate change has altered temperature and precipitation patterns, increasing the frequency of extreme events and threatening regional biodiversity [19,20,21]. The IPCC reports that species have generally migrated poleward and upward in elevation over the past century. However, some species may face population decline or extinction due to insufficient migration rates, leading to habitat fragmentation or contraction [15,22]. Given the projected continuation of climate change, understanding its impacts on species distributions is essential for biodiversity conservation and ecosystem management.

Species distribution models (SDMs) predict suitable habitats by correlating species occurrence data with environmental variables, projecting ecological niches across landscapes [23,24,25]. Integrating SDMs with climate scenarios has become a prominent approach in global change and plant ecology [17,21,26,27]. The Biomod2 platform in R enables the development of ensemble models by combining multiple algorithms, mitigating individual model biases through weighted averaging [28,29,30].

In this study, we aim to (1) project changes in *M. esculenta*’s suitable habitats under different climate scenarios; (2) analyze shifts in its ecological niche; and (3) develop a suitability–productivity model to delineate potential cultivation regions in the upper reaches of the Dadu and Min Rivers. Our findings will provide a theoretical basis for the sustainable exploitation and management of this species in the region.

## 2. Materials and Methods

### 2.1. Sample Data Collection and Filtering

**Figure 1:**
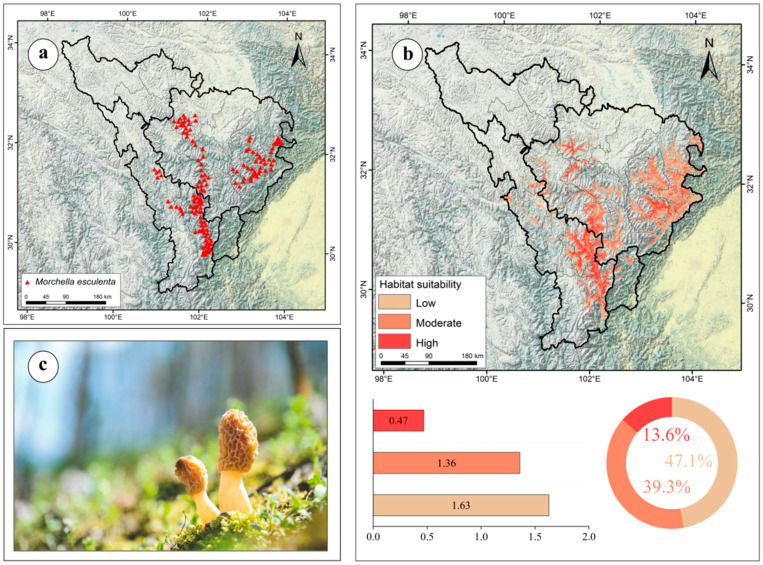
(**a**) Distribution records of *M. esculenta* in the upper reaches of the Dadu and Min Rivers; (**b**) current potential distribution areas of *M. esculenta*; (**c**) wild *M. esculenta* photographed in the field.

From 2022 to 2024, our research team conducted field surveys on *M. esculenta* in the upper reaches of the Dadu and Min Rivers, acquiring 149 valid distribution records. Using ArcGIS software (v10.2), we documented the geographic coordinates (latitude/longitude) of all *M. esculenta* occurrence points. Through the “subset” and “clean_coordinates” functions in CoordinateCleaner (v2.0), bias correction was applied to the dataset, with only one distribution point retained per 1 km × 1 km grid. A total of 120 valid sample points were obtained (Figure 1a) [31].

### 2.2. Selection and Processing of Environmental Variables

A total of 41 environmental variables were included, comprising 19 bioclimatic factors, 16 soil factors, 3 topographic factors, 1 human footprint factor, 1 land use factor, and 1 NDVI factor. Contemporary and future climate data were downloaded from the WorldClim database (http://worldclim.org/data/index.html, accessed on 1 August 2023) [32]. Three future climate scenarios (SSP126, SSP245, and SSP585) were applied, representing low-, medium-, and high-greenhouse-gas-emission pathways, respectively [33]. Soil and topographic factors were derived from the United Nations Food and Agriculture Organization’s Harmonized World Soil Database (HWSD) (http://www.fao.org/faostat/en/#data, accessed on 1 August 2023) [34]. Human footprint data (2009) were obtained from NASA’s Socioeconomic Data and Applications Center (SEDAC), which integrates eight variables: built environment, population density, electrical infrastructure, cropland, pasture, roads, railways, and navigable waterways [35]. The normalized difference vegetation index (NDVI) was retrieved from the Land Processes Distributed Active Archive Center (LPDAAC) at the USGS Earth Resources Observation and Science Center (EROS, http://LPDAAC.usgs.gov, accessed on 1 August 2023). Land use data were sourced from the Resource and Environmental Science Data Center of the Chinese Academy of Sciences (http://www.resdc.cn/Default.aspx, accessed on 1 August 2023).

To avoid collinearity among environmental variables and prevent model overfitting, VIF (variance inflation factor) screening, PCA (principal component analysis), and Spearman’s correlation tests were performed in R to improve the accuracy of ecological niche models by reducing complexity [28]. Initially, factors with correlation coefficients <0.7 were selected. Further screening retained factors with VIF values <5 [18]. For the Spearman tests, factors with correlation coefficients >0.7 were excluded, except for those with higher ecological significance. Sixteen environmental variables were ultimately selected (Table S1).

### 2.3. Ensemble Model Construction and Implementation

This study used biomod2 (v4.2.5) to develop an ensemble model, which requires species presence data and pseudo-absence data. Using the “random” method provided by biomod2, 1290 pseudo-absence points were generated from background environmental data for model simulation. The biomod2_tuning function was used to randomly select 75% of the sample points as the training dataset and the remaining 25% for model validation. Equal weights were assigned to presence and pseudo-absence data. With 10 repetitions, a total of 100 simulated models were generated [29]. Using weighted averaging, only models with a true skill statistic (TSS) ≥ 0.7 were retained to construct the ensemble model [36]. Model accuracy was evaluated using the area under the curve (AUC), kappa statistic, and TSS [31]. A 0/1 threshold (cutoff) was applied to distinguish non-suitable habitats (below threshold) from suitable habitats (above threshold), which were further divided into low-, medium-, and high-suitability tiers. The distribution change between the binary SDM tools in the ArcGIS plugin SDM tools was used to calculate niche area changes across different periods.

### 2.4. Spatial Pattern Changes in Suitable Distribution Areas

Binary suitability results (0/1) were generated using the binary.meth function in biomod2. The biomod2_RangeSize function was applied to quantify spatial pattern changes in suitable habitats for *M. esculenta* under future climate scenarios. Matrix results were visualized in ArcGIS to map distribution shifts.

### 2.5. Multivariate Environmental Similarity Surfaces (MESS) and Divergence Analysis

Using the study area’s environmental variables as reference layers, MESS and dissimilarity variable analysis were employed to identify climate anomalies and key drivers of distribution changes under future scenarios [37]. MESS evaluates the similarity of climate conditions at specific time points to the reference layer (S) [38]. A lower S value (>0) indicates greater climatic dissimilarity, while S = 100 signifies complete consistency. Values of S < 0 indicate at least one climate variable outside the reference range, signaling extreme climate change [39]. Eight environmental variables from the current suitable habitats of *M. esculenta* were used as the reference layer. This analysis was performed using the density.tools.Novel tool in Maxent (v3.4.4)’s maxent.jar package, with visualization completed in ArcGIS (v10.2) via command-line execution of the tool.

### 2.6. Niche Differentiation Analysis

Ecological niche differentiation and its environmental drivers for *M. esculenta* were quantified. Using distribution points and climate data across scenarios, the ecospat package was used to calculate niche overlap rates and visualize changes under current and future climates. The niche parameter D (observed value, ranging from 0 to 1, where 0 = no overlap, 1 = complete overlap) was calculated to assess climate impacts on niche dynamics [40]. Niche breadth in geographic and environmental space was quantified using average Levins’ B1 (inverse concentration) and B2 (uncertainty) values from habitat suitability maps, computed with ENMTools v1.3 [26]. Levins’ B1 and B2 range from 0 (narrow niche) to 1 (broad niche).

### 2.7. Centroid Migration Patterns

Centroid coordinates of suitable habitats for *M. esculenta* across periods and climate scenarios were calculated using the SDMTool package (v1.1-21) in R (Equation 4-1). Latitude/longitude coordinates and migration distances between centroids were computed in ArcGIS, with results visualized to map migration trends in the upper Dadu–Min River region.

### 2.8. Modeling Cultivation Productivity–Environmental Suitability Relationships

Through expert consultation and interdisciplinary collaboration, a cultivation dynamics model integrating ecological suitability (S) and nutrient distribution (N) was developed as P = S + N. Ecological suitability values (S) were derived from species distribution model outputs via spatial interpolation, with weights assigned based on model reliability. Nutrient indices (N) were weighted using the entropy method, with detailed weighting procedures and nutrient types described in Supplementary Text S1. After standardizing indicators (Table S2), a weighted summation was performed. Model validation used the ggtrendline package in R (v4.1.2) to fit seven nonlinear regression models to quantify relationships between cultivation productivity and ecological suitability (Table S3). The Akaike information criterion (AIC) was used to select the best model (ΔAIC < 2), which was applied to predict potential cultivation areas under current and future climates [41].

## 3. Results

### 3.1. Multi-Model Prediction Outcomes and Ensemble Model Accuracy Validation

**Figure 2:**
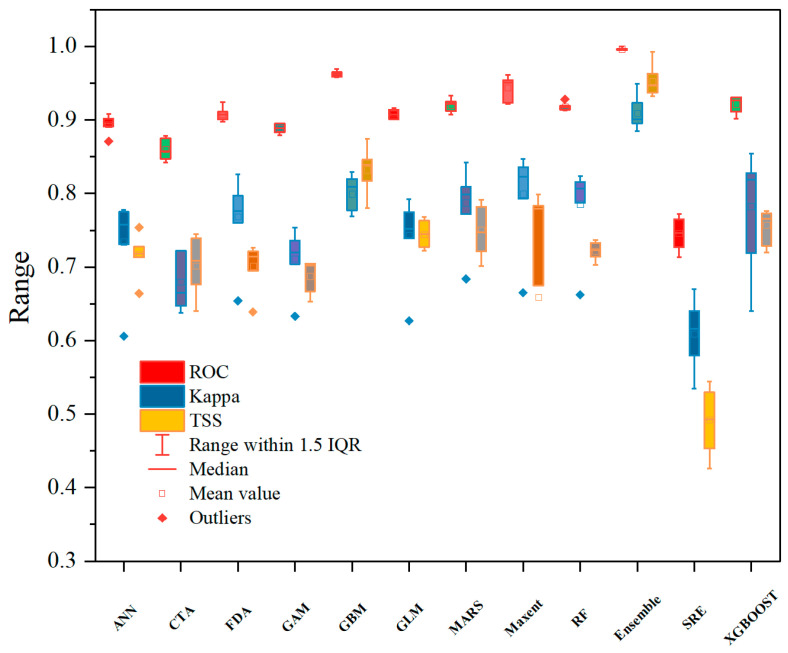
Evaluation of model accuracy based on three metrics.

The simulations of *M. esculenta* by individual models showed a broad potential distribution across the upper Dadu and Min River regions, excluding only its northwestern areas. This may be attributed to the complex topography of the upper Dadu River–Minjiang River basin, where deep alpine valleys create steep elevational gradients and pronounced microclimatic variation. Within each valley system, these conditions generate discrete elevational belts exhibiting optimal ecological suitability for *M. esculenta* growth. While all models agreed on the general trend, significant discrepancies existed in predictive details (Figure S1). The biomod_tuning function optimized model parameters, with performance evaluated using receiver operating characteristic (ROC), kappa, and true skill statistic (TSS) metrics in each iteration. The ensemble model achieved a mean TSS of 0.940, ROC of 0.994, and kappa of 0.873, significantly outperforming individual models (Figure 2). These results indicate that the ensemble model provided the best fit and most reliable predictions.

### 3.2. Environmental Factor Combinations Influencing M. esculenta Potential Distribution

The current literature lacks consensus on determining the number of dominant environmental factors, with most studies relying on contribution rates—though subjective threshold selection remains an issue. Here, the top three contributing factors were selected as the dominant combination. The calculations identified temperature annual range (Bio7, 13.10%), precipitation of the driest month (Bio14, 8.47%), and temperature seasonality (Bio4, 18.47%) as the primary limiting factors, with a cumulative contribution rate of 40.04% (Figure S2).

### 3.3. Multivariate Environmental Similarity Surfaces (MESS) and Most Dissimilar Variables (MOD) Analysis

Based on factor contributions from the ensemble model (Figure S2), eight environmental variables with >10% contribution—annual mean temperature (bio1), temperature seasonality (bio4), max temperature of the warmest month (bio5), min temperature of the coldest month (bio6), temperature annual range (bio7), annual precipitation (bio12), precipitation of the driest month (bio14), and gravel content (t_gravel)—were selected for MESS and MOD analysis.

**Figure 3:**
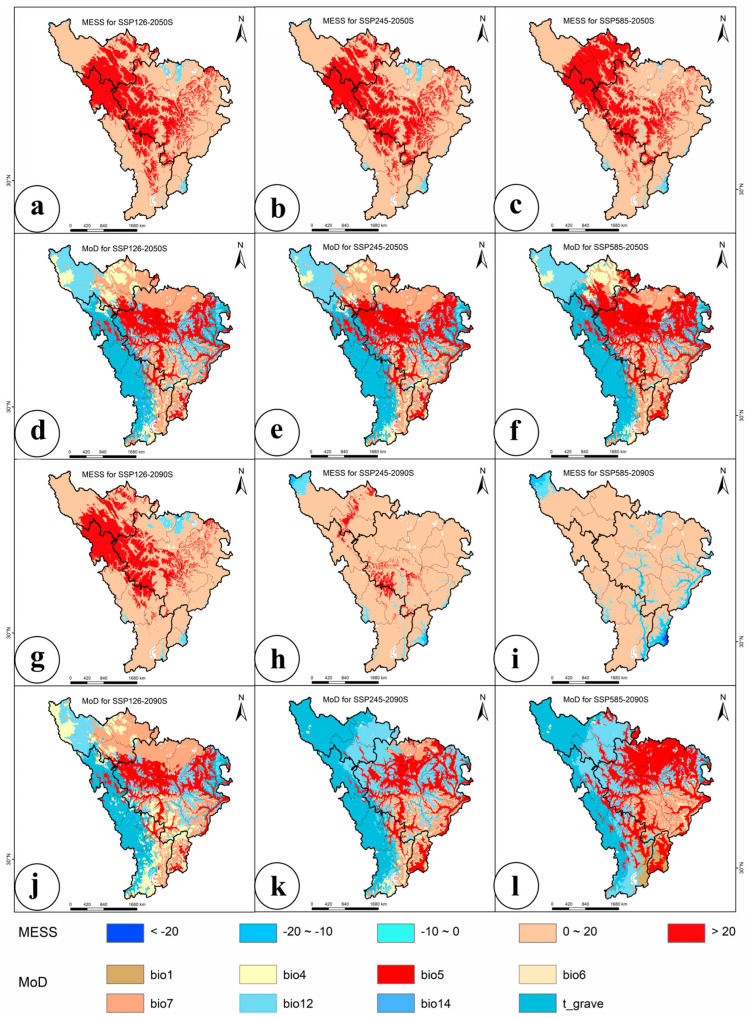
Analysis of MESS and MOD. (**a**) MESS for 2050s-SSP1-2, (**b**) MESS for 2050s-SSP2-4.5, (**c**) MESS for 2050s-SSP5-8.5, (**d**) MOD for 2050s-SSP1-2.6, (**e**) MOD for 2050s-SSP2-4.5, (**f**) MOD for 2050s-SSP5-8.5, (**g**) MESS for 2090s-SSP1-2.6, (**h**) MESS for 2090s-SSP2-4.5, (**i**) MESS for 2090s-SSP5-8, (**j**) MOD for 2090s-SSP1-2.6, (**k**) MOD for 2090s-SSP2-4.5, (**l**) MOD for 2090s-SSP5-8.5.

Under different climate scenarios, the average MESS values for modern distribution points were 36.1, 29.8, 29.3, 37.2, 24.6, and 4.1 by the mid- and late 21st century, respectively (Figure 3). The lowest MESS value (4.1) occurred under the SSP5-8.5 (high-emission) scenario in 2090, indicating that severe climate warming could disrupt hydrothermal regimes (e.g., extreme precipitation or temperature increases), threatening ecosystem stability. Higher MESS values under SSP1-2.6 (low-emission) scenarios suggested that low-carbon pathways maintain environmental similarity (Figure 3). Collectively, these results highlight significant climate anomalies and the high vulnerability of *M. esculenta* distribution to future climate change in this region.

### 3.4. Current Potential Distribution of M. esculenta in the Upper Dadu–Min River Region

The current potential distribution of *M. esculenta* is shown in Figure 1b, with a total suitable habitat area of 3.46 × 10^4^ km^2^. High-suitability habitats (0.47 × 10^4^ km^2^, 13.60% of total) formed three major clusters: the largest in Danba County, southern Jinchuan County, and northeastern Kangding City; two smaller clusters in northeastern Lixian County, southwestern Aba County, and northwestern Maerkang City; and sporadic distributions in Heishui, Maoxian, and Wenchuan Counties (Figure 1b). Medium-suitability habitats (1.36 × 10^4^ km^2^, 39.30%) surrounded high-suitability areas and occurred as discrete patches in Xiaojin and Luhuo Counties (Figure 1b).

### 3.5. Future Potential Distribution of M. esculenta in the Upper Dadu–Min River Region

**Figure 4:**
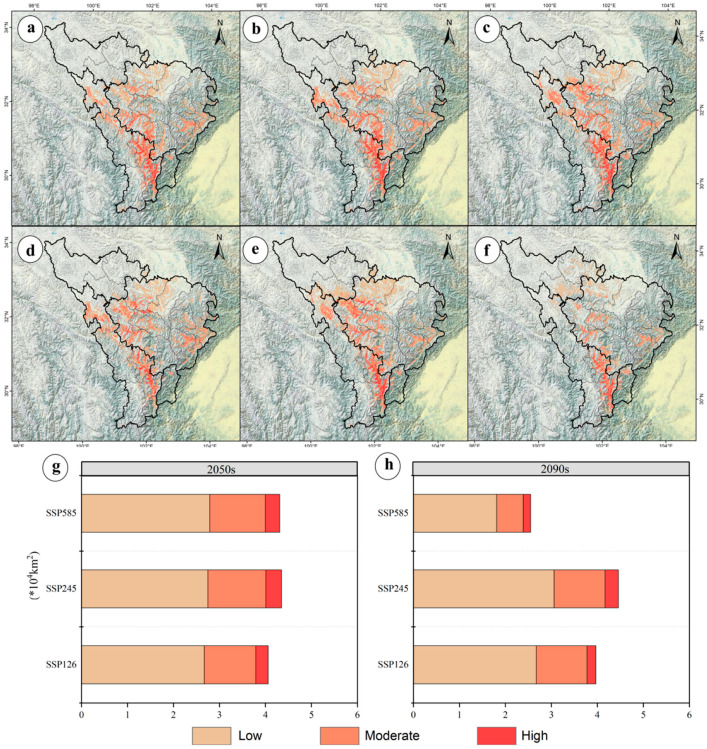
Future potential distributions of *M. esculenta* in the upper Dadu and Min River region under climate change scenarios. Potential distributions under SSP1-2.6 (**a**,**d**), SSP2-4.5 (**b**,**e**), and SSP5-8.5 (**c**,**f**) for the 2050s (**a**–**c**) and 2090s (**d**–**f**). Areal changes in three suitability tiers under different scenarios for the 2050s (**g**) and 2090s (**h**).

Using the ensemble model, potential distributions under SSP1-2.6, SSP2-4.5, and SSP5-8.5 scenarios were projected for 2050 and 2090 (Figure 4). Total suitable habitat area increased most under SSP2-4.5 in 2050 (+25.72%, 0.89 × 10^4^ km^2^) but decreased most under SSP5-8.5 in 2090 (−26.30%, 0.91 × 10^4^ km^2^) (Figure 4). High-suitability habitats declined most under SSP5-8.5 in 2090 (−65.96%, 0.31 × 10^4^ km^2^), with no increases observed in any scenario; the smallest decline occurred under SSP2-4.5 in 2050 (−27.66%, 0.13 × 10^4^ km^2^) (Figure 4). Medium-suitability habitats declined most under SSP5-8.5 in 2090 (−57.35%, 0.78 × 10^4^ km^2^) and least under SSP2-4.5 in 2050 (−7.35%, 0.10 × 10^4^ km^2^) (Figure 3). Low-suitability habitats increased most under SSP2-4.5 in 2050 (+68.71%, 1.12 × 10^4^ km^2^) and declined least under SSP5-8.5 in 2090 (−11.04%, 0.18 × 10^4^ km^2^) (Figure 4). These trends indicate a shift toward “dominated by low-suitability expansion and continuous high-suitability contraction.”

### 3.6. Future Potential Distribution Patterns of M. esculenta in the Upper Dadu–Min River Region

**Figure 5:**
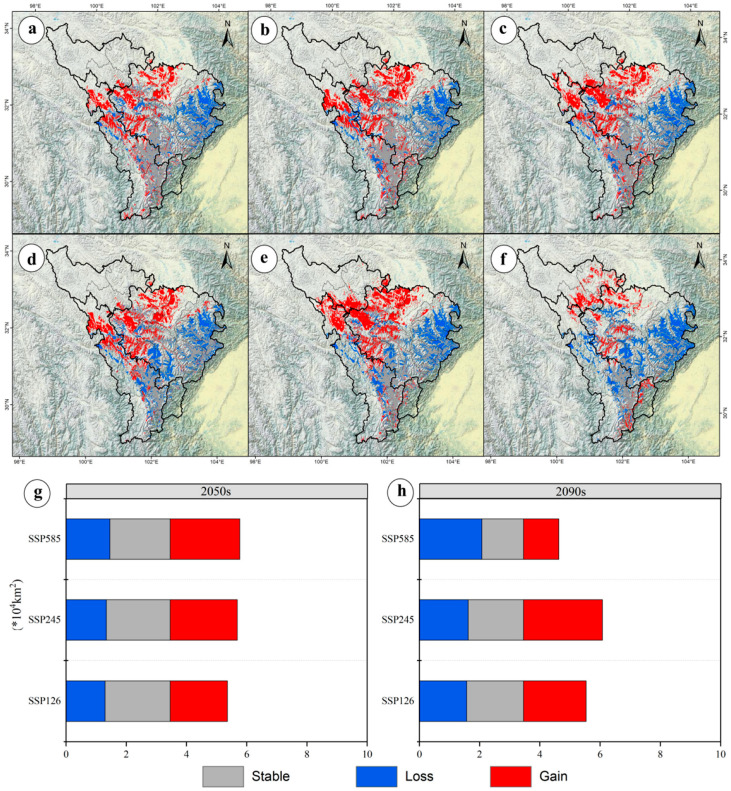
Changes in potential distribution areas of *M. esculenta* in the upper Dadu and Min River region under climate change. Distribution changes under SSP1-2.6 (**a**,**d**), SSP2-4.5 (**b**,**e**), and SSP5-8.5 (**c**,**f**) for the 2050s (**a**–**c**) and 2090s (**d**–**f**). Areal changes in suitable habitats under different scenarios for the 2050s (**g**) and 2090s (**h**).

Using the ensemble model, potential distributions of *M. esculenta* in the upper Dadu and Min River region were predicted for 2050 and 2090 under the SSP1-2.6, SSP2-4.5, and SSP5-8.5 scenarios (Figure 4). Spatial overlay analysis in ArcGIS revealed changes in potential distributions of key wild herbivorous resources under future climate scenarios (Figure 5). As shown in Figure 5, under future climate scenarios, the suitable habitat area for M. esculenta will expand significantly compared to the current situation. This expansion is mainly concentrated in Aba County, Zangtang County, Seda County, and Luhuo County, indicating that its distribution pattern shows a clear northward migration trend. In the 2050 period, the largest expansion occurred under SSP2-4.5 (+64.46%, 2.23 × 10^4^ km^2^), while the smallest expansion occurred under SSP1-2.6 (+54.84%, 1.90 × 10^4^ km^2^) (Figure 5). By the 2090 period, expansion continued to lead under SSP2-4.5 (+75.58%, 2.62 × 10^4^ km^2^), whereas, under SSP5-8.5, suitable habitats contracted by 33.74%, leaving only 1.17 × 10^4^ km^2^ (Figure 5). These results indicate that *M. esculenta* distribution performed best under the medium-emission scenario.

### 3.7. Analysis of Niche Shifts in M. esculenta Under Future Climates

**Figure 6:**
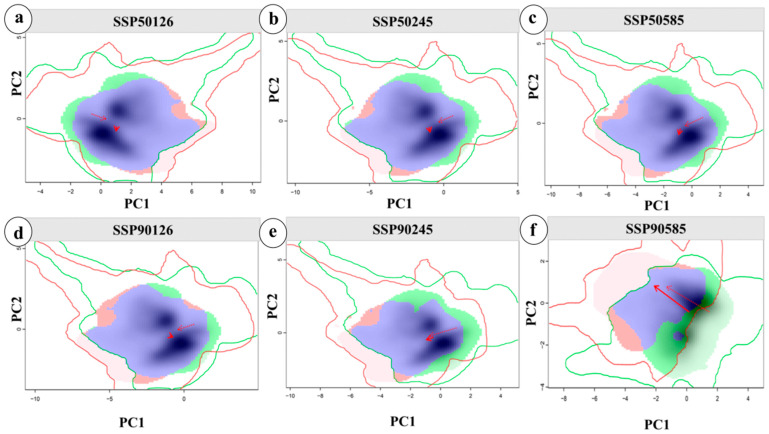
Niche shifts of *M. esculenta* under climate change. Niche changes under SSP1-2.6 (**a**,**d**), SSP2-4.5 (**b**,**e**), and SSP5-8.5 (**c**,**f**) for the 2050s (**a**–**c**) and 2090s (**d**–**f**). Green and red shading indicate species occurrence density in current and future scenarios, respectively; blue represents overlap. Solid and dashed lines denote 100% and 50% of available environmental space. Red arrows mark how the climatic niche centroid (solid line) and background range centroid (dashed line) shift between periods.

We quantified niche differentiation and environmental drivers of *M. esculenta* under SSP1-2.6, SSP2-4.5, and SSP5-8.5 scenarios for 2050 and 2090. Using distribution points and climate data across scenarios, the ecospat package was used to calculate niche overlap rates and visualize niche changes. The niche overlap of *M. esculenta* in the upper Dadu–Min River region is shown in Figure 6. Niche overlap rates decreased significantly under SSP5-8.5, with D values of 0.661 in the 2050s and 0.313 in the 2090s, indicating accelerated niche differentiation and lower niche equivalence, potentially driving migration toward high-elevation cold, wet areas under high-emission scenarios. Principal component analysis (PCA) showed that the first two principal components explained 68.69–72.10% of environmental factor variance (PC1: 51.53–53.31%; PC2: 17.16–18.79%), with temperature annual range, precipitation of the driest month, and temperature seasonality identified as primary drivers of niche shifts. The future climatic niche centroid is projected to shift toward areas with higher temperature annual range and temperature seasonality.

### 3.8. Centroid Migration Trajectories Under Climate Change

**Figure 7:**
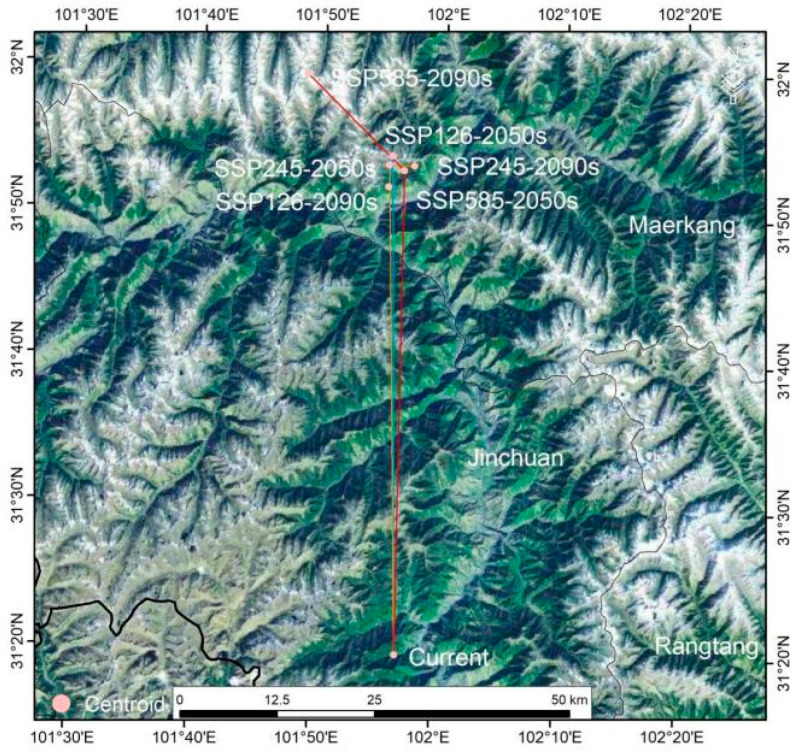
Variations in the centroids of suitable areas of *M. esculenta* under climate change scenarios.

Based on the centroids of *M. esculenta* suitable habitats under current and future climate scenarios, their movement trajectories and trends were analyzed. The centroid of suitable habitats for contemporary *M. esculenta* in the upper Dadu–Min River region is located at 101.9503 E/31.3312 N (Figure 7). Under the SSP1-2.6 scenario, the centroid first moved 63.17 km northward to (101.9282 E, 31.9006 N) in the 2050s, then continued shifting 3.92 km southwest to (101.9238 E, 31.8655 N) by the 2090s (Figure 7). Under SSP2-4.5, the centroid migrated 62.06 km north-northwest to (101.9242 E, 31.8904 N) in the 2050s, followed by a 3.31 km southeast shift to (101.9592 E, 31.8896 N) in the 2090s (Figure 7). Under SSP5-8.5, the centroid moved 59.29 km north-northeast to (101.9451 E, 31.8841 N) in the 2050s, then shifted 17.74 km northwest to (101.8086 E, 31.9917 N) by the 2090s (Figure 7). Overall, across all three emission scenarios, the centroid of suitable habitats exhibited a northward migration trend from the baseline period to 2050 and onward to 2090.

### 3.9. Dynamics of Potential Cultivation Production Areas Across Periods

**Figure 8:**
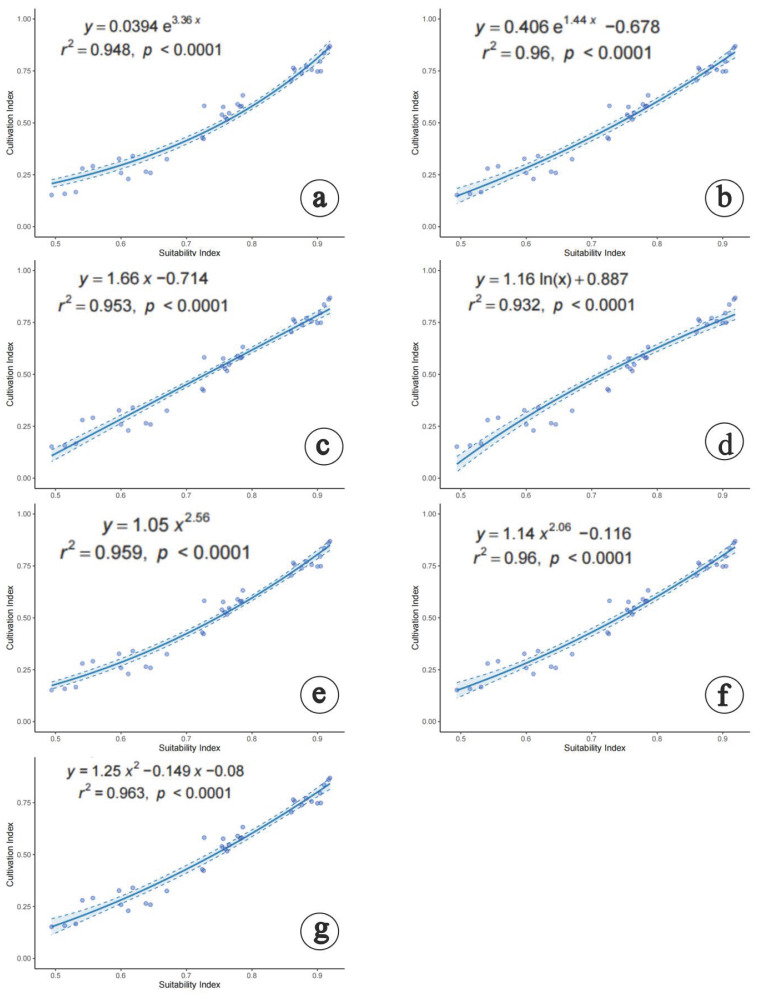
Relationships between suitability and productivity for *M. esculenta*. (**a**) exp2P model, (**b**) exp3P model, (**c**) line2P model, (**d**) log2P model, (**e**) power2P model, (**f**) power3P model, (**g**) line3P model.

According to the Akaike information criterion (AIC), a significant positive correlation was observed between *M. esculenta* habitat suitability and productivity across seven regression models (Figure 8). The line3P model (R^2^ = 0.963) was identified as the optimal model, followed by the exp3P and power3P models (R^2^ = 0.960), power2P (R^2^ = 0.959), line2P (R^2^ = 0.953), exp2P (R^2^ = 0.948), and log2P (R^2^ = 0.932) models. Thus, the line3P model was selected to construct the productivity–suitability model (Figure 8). The cultivation areas delineated in this study based on the production-driven model represent areas suitable for cultivation under natural environmental constraints.

**Figure 9:**
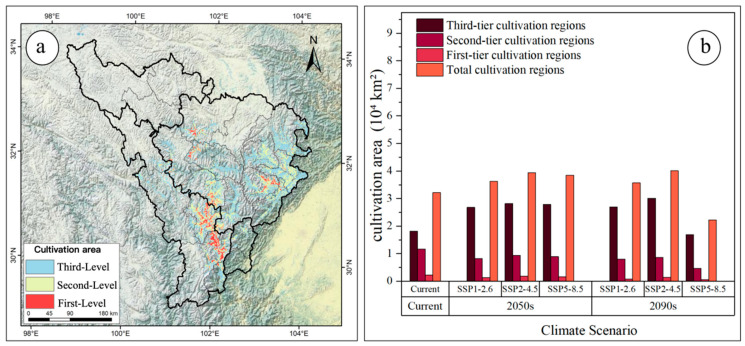
(**a**) Current cultivation areas of *M. esculenta*; (**b**) changes in cultivation area across different periods.

**Figure 10:**
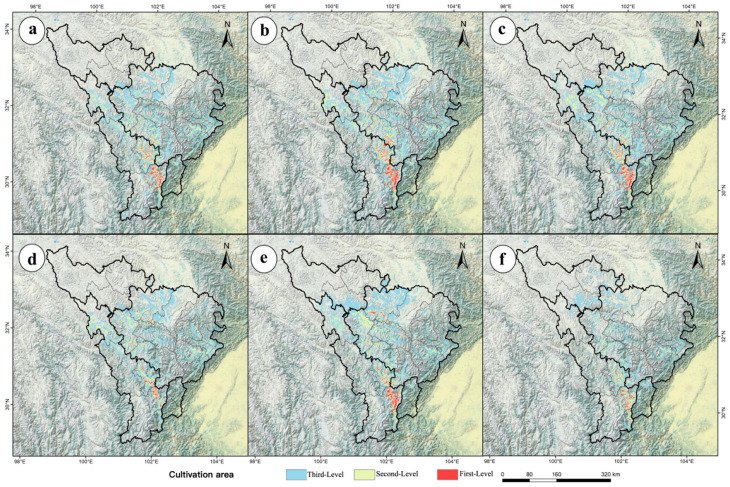
Distribution of *M. esculenta* cultivation tiers in the upper Dadu and Min River region under future climate scenarios. Cultivation tier distributions under SSP1-2.6 (**a**,**d**), SSP2-4.5 (**b**,**e**), and SSP5-8.5 (**c**,**f**) for the 2050s (**a**–**c**) and 2090s (**d**–**f**).

Using the production dynamics model, productivity was classified into three tiers: high (>0.58), medium (0.37–0.58), and low (<0.37). High-productivity areas were designated as first-tier cultivation regions (core zones), medium-productivity areas as second-tier cultivation regions (general zones), and low-productivity areas as third-tier cultivation regions (peripheral zones) (Figure 9a and Figure 10).

Under current climate conditions, the total cultivation area is 3.22 × 10^4^ km^2^, including 0.23 × 10^4^ km^2^ of first-tier cultivation areas and 1.17 × 10^4^ km^2^ of second-tier cultivation areas (Figure 9b). Current cultivation areas of *M. esculenta* are primarily concentrated in the entire upper Min River and parts of the upper Dadu River, with first-tier areas mainly distributed in Kangding City, Danba County, Maerkang City, Lixian County, and surrounding areas (Figure 9a). Quantitative analysis of spatiotemporal changes in the cultivation areas under different shared socioeconomic pathways (SSPs) revealed significant shifts across the three cultivation tiers (Figure 10). Compared to the present, the future climate scenarios showed consistent trends: first- and second-tier cultivation areas declined, while third-tier (peripheral) areas expanded with increasing emission intensity or time (Figure 9b). In the 2050 period, the total cultivation areas increased under all three scenarios, though the first- and second-tier areas decreased. The largest total area expansion occurred under SSP2-4.5 (+3.94 × 10^4^ km^2^), with the first-tier areas declining to 0.18 × 10^4^ km^2^ and the second-tier to 0.94 × 10^4^ km^2^ (Figure 6b). By the 2090 period, the total cultivation areas continued to increase under SSP1-2.6 and SSP2-4.5 but contracted drastically under SSP5-8.5. The maximum expansion under SSP2-4.5 reached 4.01 × 10^4^ km^2^, with the first-tier and second-tier areas dropping to 0.18 × 10^4^ km^2^ and 0.86 × 10^4^ km^2^, respectively (Figure 9b). Under SSP5-8.5 in 2090, all the cultivation tiers declined sharply: total area fell to 2.22 × 10^4^ km^2^, first-tier to 0.06 × 10^4^ km^2^, and second-tier to 0.47 × 10^4^ km^2^ (Figure 9b). Overall, the future climate scenarios indicate a trend of expanding total and third-tier cultivation areas alongside shrinking first- and second-tier areas, suggesting substantial conversion of high-quality cultivation zones to peripheral areas and transformation of unsuitable lands into marginal cultivation zones.

## 4. Discussion

Climate change may accelerate species extinction, reduce biodiversity, and render regional ecosystems more vulnerable, while some species may develop new physiological traits to adapt [15,16,17,18,26,27]. Over recent decades, research on global climate and environmental change has gained increasing prominence in scientific inquiry [19,20,42]. Global climate change is reshaping terrestrial ecosystem patterns at an unprecedented rate. The IPCC Sixth Assessment Report notes that global surface temperatures have risen at a rate of 0.2 °C per decade since 1970, directly causing latitude or elevation shifts in the distribution ranges of over 50% of plant species [22]. Such changes are particularly pronounced in mountain ecosystems, where altered temperature and precipitation patterns not only compress the survival space of endemic species but also influence resource allocation and population adaptability through niche shift mechanisms [43]. To quantify the potential impacts of climate change on *M. esculenta* distribution, this study executed a four-phase analytical framework. First, twelve widely employed species distribution models underwent comparative analysis, with the optimal model selected for the study area through a tripartite evaluation system. Second, environmental predictors were refined sequentially, starting with collinearity diagnosis to screen modeling variables, followed by contribution rate analysis to identify the dominant factors limiting the potential distribution, and finally ending with multivariate environmental similarity surface (MESS) and least similar variable (MOD) analysis to quantify the magnitude of future climate anomalies. Third, the optimized model processed these variables to project contemporary and future potential distributions of Morchella esculenta, including niche dynamics and habitat centroid migration trajectories. Fourth, standardized nutritional data provided information for delineating production dynamics models for natural growing areas. The comprehensive workflow detailed in Figure S3 laid the theoretical foundation for the sustainable resource development of *M. esculenta* in this region.

Based on current distribution patterns, *M. esculenta* exhibits broad prevalence across the study area, with potential habitats encompassing virtually all sectors except the northwestern portion of the upper Dadu River–Minjiang River region. Three core distribution centers of high suitability were identified within this region. This spatial configuration is attributable to the basin’s characteristic alpine valley topography, which generates substantial elevational gradients and pronounced climatic heterogeneity, thereby establishing discrete optimal growth zones within each river corridor.

The northward migration of plant habitat centroids to higher latitudes or elevations under climate change has been confirmed by numerous classic studies. Parmesan and Yohe [43], by synthesizing global species distribution data, found that 80% of species exhibited poleward or upward elevation shifts due to warming, with temperate plants showing particularly strong responses to temperature changes. This pattern has been validated across multiple Eurasian ecosystems: Lenoir et al. [44] analyzed 171 plant species in six European mountain ranges and found that plant communities shifted upward by an average of 29 m per decade over the past century, highly synchronized with regional warming rates. In East Asia, Zhu et al. [45], based on distribution data of 2468 Chinese plant species, found that 53% of species exhibited significant northward shifts in their climatic niches, with alpine/subalpine plants in the Hengduan Mountains migrating at rates far exceeding low-elevation species, confirming differential climate change impacts on mountain ecosystems. Climate warming typically affects species’ potential geographic distributions through range shifts to higher latitudes/elevations and area expansions/contractions [46,47,48]. Our finding that *M. esculenta*’s potential suitable habitats will shift northward to higher latitudes under future climate scenarios aligns with this “climate-driven niche shift” mechanism revealed in classic cases, further supporting the latitude adaptation strategy of Northern Hemisphere plants to climate warming.

Regarding *M. esculenta*’s niche dynamics, pairwise comparisons between current and future scenarios showed decreasing niche overlap with increasing climate change severity. Temperature annual range (Bio7), precipitation of the driest month (Bio14), and temperature seasonality (Bio4) were identified as primary drivers of niche differentiation (Figure S2). Under ongoing global warming, temperature changes in high-altitude regions like Tibet and Qinghai have been drastic [49]. The upper Dadu–Min River region, located on the eastern edge of the Tibetan Plateau [12], is naturally influenced by these changes, with local plants undoubtedly affected by such thermal fluctuations. The results showed that while the total suitable habitats and cultivation areas for *M. esculenta* are projected to increase under climate change, high-suitability habitats and first-tier cultivation areas will decline sharply, revealing the nonlinear responses of its suitable habitats to climate change. This contrasts with migration patterns of higher plants in the Northern Hemisphere—for example, European beech (*Fagus sylvatica*) expands poleward at 30–50 km per decade under warming, with simultaneous increases in distribution area and habitat quality [50]. The divergent response of *M. esculenta* may stem from its fungal biology: when the temperature annual range (Bio7) exceeds its suitable threshold, mycelial dehydrogenase activity decreases significantly, blocking nutrient accumulation [8]; insufficient precipitation in the driest month (Bio14) causes dehydration and apoptosis of fruiting body primordia [51]. These findings highlight the higher climate vulnerability of saprophytic fungi compared to higher plants. Additionally, Pauli et al. [52] documented a “mountain-top trap” effect in alpine ecosystems under warming, where species face limited migration options due to elevation limits. *M. esculenta* in alpine areas likely experiences this trap, leading to a polarized pattern of decreasing high/medium-suitability habitats and high-quality cultivation areas, rather than simple linear range shifts.

Projections of *M. esculenta*’s potential distribution in the upper Dadu–Min River region indicate significant reductions in high- and medium-suitability habitats under climate change, posing challenges for sustainable resource use. Therefore, this study delineates potential natural cultivation zones for *M. esculenta* using a production dynamics model, providing a scientific basis for its sustainable utilization under future climate scenarios. Current cultivation areas are concentrated in the entire upper Min River and parts of the upper Dadu River (Kangding City, Daofu County, Danba County) (Figure 9a), where hydrothermal conditions align with its ecological requirements. However, future warming will substantially alter cultivation patterns: under SSP5-8.5, core cultivation areas will shrink drastically by 2090, with distributions shifting toward higher elevations and latitudes (Figure 6). This trend suggests climate change may challenge the large-scale cultivation and resource management of *M. esculenta*.

To address this polarized evolution of cultivation patterns, we propose prioritizing standardized cultivation in current core areas to meet market demands while adopting adaptive management strategies: microclimate protection in existing cores (Wenchuan-Lixian): establish 500 m radius microclimate protection zones around cultivation sites, lay ≥10 cm thick birch wood chip layers to maintain soil moisture >65%, and replant host oak trees at 50–80 stems/ha density in a 200 m buffer zone. Innovative cultivation in new marginal areas (Danba-Jinchuan under SSP2-4.5): apply the validated “wood substrate embedded cultivation” method from Oregon [53], mixing hardwood chips and humus at 1:3 to adjust the C:N ratio to 25:1 and enhance yields. Corridor conservation along centroid migration trajectories (Figure 7): establish community co-management networks to protect host plants along migration pathways and safeguard ecological corridors for *M. esculenta*.

These findings constitute an initial step for macro-scale planning, providing critical foundations for scientific management and sustainable utilization of *M. esculenta*. However, the predictive ability and stability of the model are still subject to several constraints, as sample size, spatial resolution, and the selection of climate variables themselves can affect performance. While distribution data from field surveys ensured robust model execution, potential sampling omissions cannot be entirely discounted. Future habitat projections, derived from contemporary occurrence records, carry inherent systematic errors. Crucially, *M. esculenta* distribution and growth are governed not solely by climate but also by biotic interactions (e.g., natural enemy dynamics) and multifaceted environmental determinants, including vegetation types and socioeconomic structures. Given the inherent unpredictability of future variables, practical applications must comprehensively evaluate these confounding factors within adaptive management frameworks.

## 5. Conclusions

To quantify the potential impacts of climate change on *M. esculenta* distribution, this study integrated contemporary and future (2050 and 2090) environmental factors under three emission scenarios using ensemble models to project evolution patterns of its potential distribution and cultivation regions, aiming to provide a theoretical basis for sustainable exploitation in this region. The results show that current high-suitability habitats and core cultivation regions of *M. esculenta* are predominantly in patchy and fragmented distributions. Three high-suitability centers exist in the upper Dadu and Min River region: the largest is in Danba County, southern Jinchuan County, and northeastern Kangding City; two smaller centers are in northeastern Lixian County, southwestern Aba County, northwestern Maerkang City, with sporadic distributions in Heishui, Maoxian, and Wenchuan Counties. First-tier cultivation regions are mainly concentrated in Kangding City, Danba County, Maerkang City, Lixian County, and surrounding areas. Under climate warming, both the potential distribution and cultivation areas of *M. esculenta* in this region exhibit a polarized pattern of “high-quality suitable areas contracting while low-quality ones expanding,” accompanied by gradual migration of its climatic niche and habitat centroid. To address this, sustainable utilization of *M. esculenta* requires (1) standardized cultivation in current core regions to meet market demands and (2) adaptive management strategies to dynamically adjust cultivation zones. Overall, this study not only provides decision support for sustainable *M. esculenta* resource management but also offers insights into biodiversity conservation and livelihood adaptation in alpine canyon regions under global change.

## Data Availability

The raw data of this article will be made available by corresponding authors, according to the personal requests.

## Supplementary Materials

The following supporting information can be downloaded at: https://www.mdpi.com/article/10.3390/jof11070475/s1, Text S1: Methods for Setting Weighting Ratios and Rationale for *M. esculenta* Nutritional Components; Figure S1: Current potential distribution of *M. esculenta* in the upper reaches of the Dadu and Min Rivers based on multi-model predictions. (a) ANN model, (b) GTA model, (c) FDA model, (d) GAM model, (e) GBM model, (f) GLM model, (g) MARS model, (h) Maxent model, (i) RF model, (j) SER model, (k) XGBOOST model, (l) Ensemble model; Figure S2: Environmental variables and their contributions; Figure S3: Flowchart displaying the steps of the present study; Table S1: 16 Environmental Variables Involved in Modeling; Table S2: Seven types of models used for modeling the relationship between productivity and suitability; Table S3: Standardized Results of *M. esculenta* Indicators; Table S4: Types of *M. esculenta* nutritional components and their weighting ratios.
