# Supplementary material for: Evolution of Potential Distribution Areas and Cultivation Zones of Morchella esculenta (L.) Pers. Under Climate Warming: Application of Ensemble Models and Production Dynamics Models
Source: J Fungi (Basel). 2025 Jun 22;11(7):475. doi: 10.3390/jof11070475 (PMC12295879; doi:10.3390/jof11070475)
Supplement: Supplementary file 1 [file jof-11-00475-s001.zip › jof-3709788-supplementary.pdf]

# Supplementary material

**Text S1. Methods for Setting Weighting Ratios and Rationale for *M. esculenta* Nutritional Components**

This study measured proximal nutrients, bioactive compounds, and amino acid profiles of *M. esculenta*. Through multiple workshops with experts from the Sichuan Provincial Key Laboratory of Ecology and the Sichuan Provincial Engineering Center for Disaster Prevention and Mitigation, weights were assigned to components based on their nutritional functions, health values, and environmental sensitivity (Table S4).

A total weight of 100% was allocated, with proximal nutrients and bioactive compounds accounting for 75%:

- **Protein (12%)**: As a core indicator of growth and quality, regulated by nitrogen utilization efficiency.
- **Dietary fiber (10%)**: Reflects soil and moisture conditions through cell wall component regulation, with gut health benefits.
- **Fat (7%)**, **crude fiber (5%)**, and **ash (1%)**: Allocated by energy density, stress resistance, and mineral content, respectively.
- **Starch (15%)**: A core component of carbohydrate metabolism, synthesized under light-temperature conditions via key enzymes like ADP-glucose pyrophosphorylase.
- **Total flavonoids (12%)**: Enhances antioxidant capacity via UV-B induced phenylpropanoid metabolism.
- **Total saponins (8%)** and **tannins (5%)**: Indicate immunomodulatory potential and stress response, respectively.

Amino acid profiles accounted for 25%:

- **Essential amino acids (12%)**: 1.5% per amino acid, dependent on nitrogen metabolism efficiency.
- **Conditionally essential amino acids (5%)**: Arginine (2%, involved in salt stress response), cysteine (1.5%, reflects sulfur metabolism), tyrosine (1.5%, indicates secondary metabolism regulation).
- **Glutamate (4%)**: As a nitrogen metabolism hub in salt stress regulation.
- **Other non-essential amino acids (4%)**: Proline (2%, responds to drought via osmotic adjustment), remaining amino acids (0.5% each).

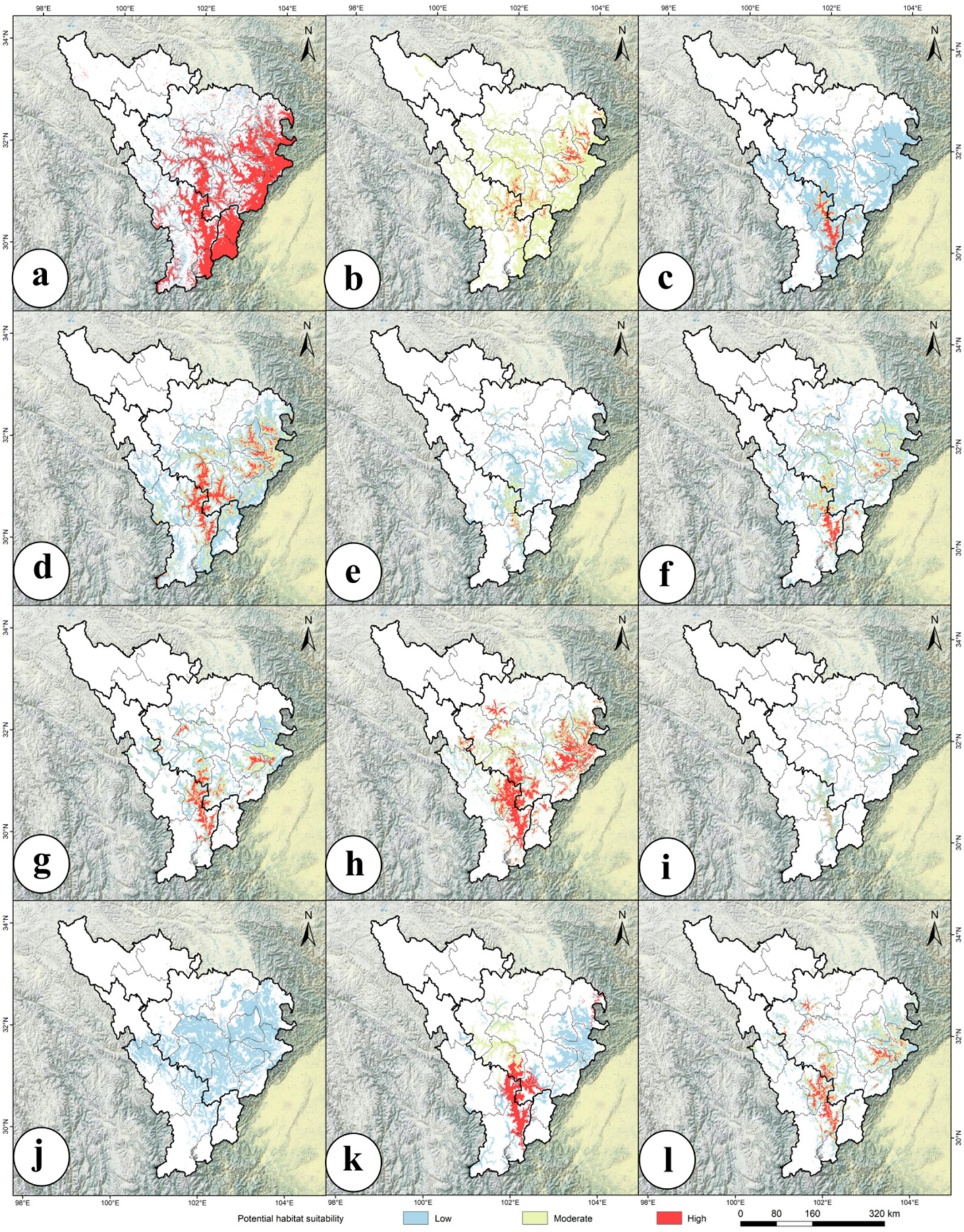


**Figure S1.**Current potential distribution of *M. esculenta* in the upper reaches of the Dadu and Min Rivers based on multi-model predictions. (a) ANN model, (b) GTA model, (c) FDA model, (d) GAM model, (e) GBM model, (f) GLM model, (g) MARS model, (h) Maxent model, (i) RF model, (j) SER model, (k) XGBOOST model, (l) Ensemble model.

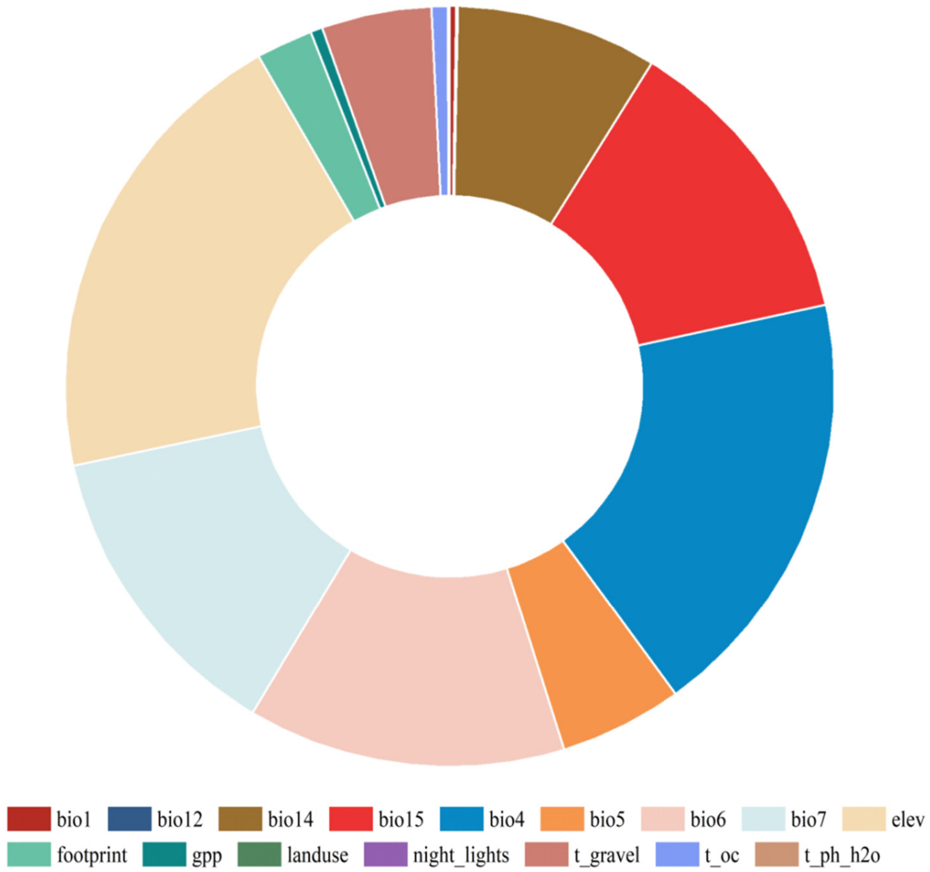


**Figure S2.** Environmental variables and their contributions.

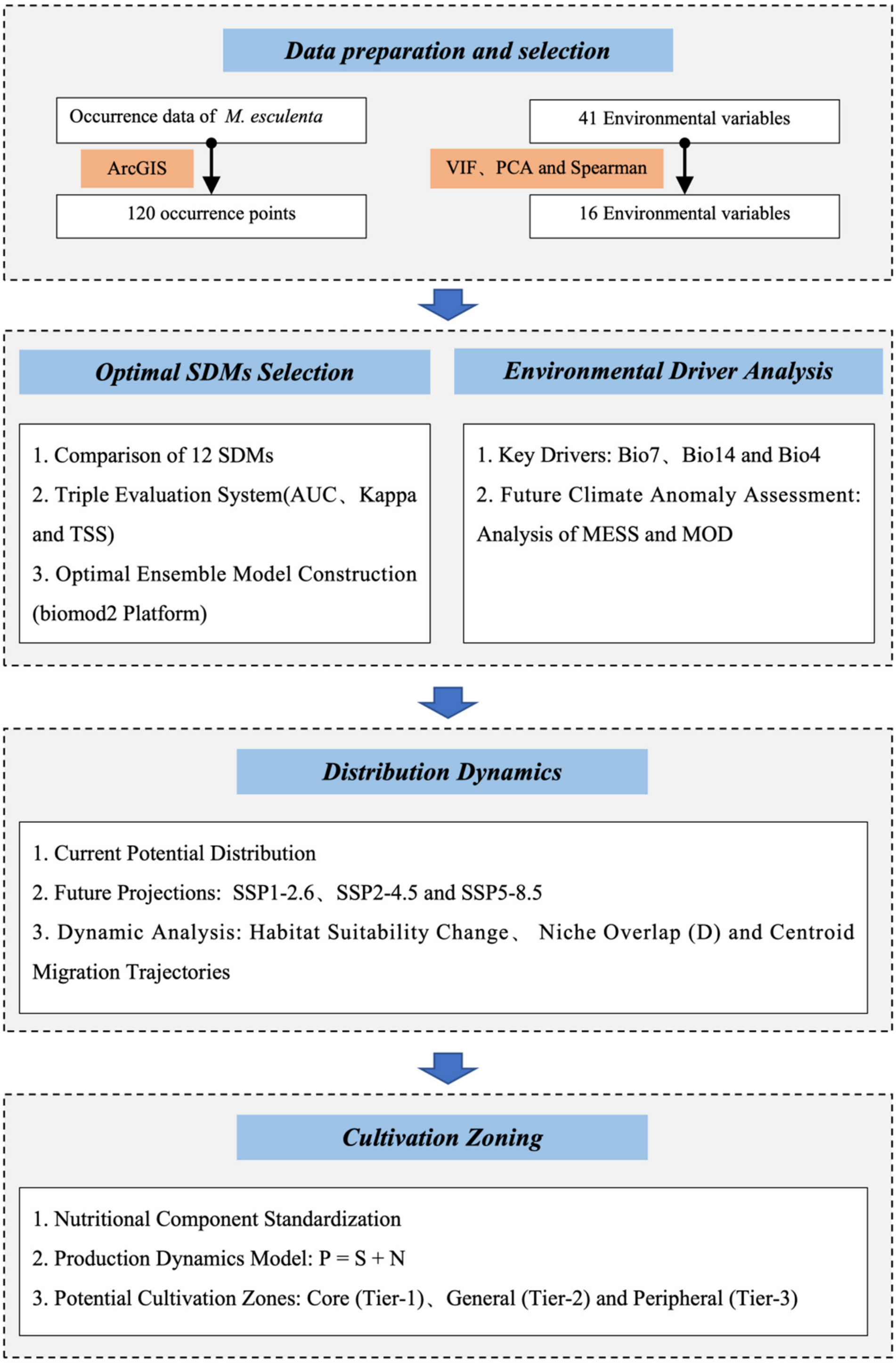


**Figure S**3. Flowchart displaying the steps of the present study.

**Table S1.** 16 Environmental Variables Involved in Modeling

| Environment variable | Abbreviation | Unit |
|---|---|---|
| Maximum temperature of warmest month | bio5 | °C |
| Minimum temperature of coldest month | bio6 | °C |
| Elevation | elev | m |
| Ecological footprint | footprint | gha |
| Land cover | landcover | / |
| Seasonal dry matter production | dmps | $g/m^2$/season |
| Gross primary productivity | gpp | g $C/m^2$/year |
| Annual mean temperature | bio1 | °C |
| Annual precipitation | bio12 | mm |
| Temperature seasonality (Coefficient of Variation) | bio4 | C of V |
| Annual temperature range | bio7 | °C |
| Precipitation of driest month | bio14 | mm |
| Precipitation seasonality (Coefficient of Variation) | bio15 | C of V |
| Gravel content | t_gravel | / |
| Soil organic carbon content | t_oc | % |
| Soil pH (water extract) | t_ph_h2o | / |

**Table S2.** Seven types of models used for modeling the relationship between productivity and suitability

| Model code | Model type |
|---|---|
| exp2P | y=a*exp(b*x) |
| exp3P | y=a*exp(b*x)+c |
| line2P | y = a*x + b |
| line3P | y=a*x^2+b*x+c |
| log2P | y=a*ln(x)+b |
| power2P | y=a*x^b |
| power3P | y=a*x^b+c |

## **Table S3.** Standardized Results of *M. esculenta* Indicators

| | | | Conventional nutritional components | | | | | | Active ingredient | | | Amino acid composition | | | | | | | | | | | | | | | | |
|---|---|---|---|---|---|---|---|---|---|---|---|---|---|---|---|---|---|---|---|---|---|---|---|---|---|---|---|---|
| Longitude | Latitude | *S*uitability | CFT | CFR | CAH | CPN | DFR | STH | SAN | FLD | TAN | ASP | THR | SER | GLU | GLY | ALA | PRO | CYS | VAL | MET | ILE | LEU | TYR | PHE | HIS | LYS | ARG |
| 101.801 | 30.894 | 0.87 | 5.24 | 3.94 | 0.80 | 11.83 | 9.86 | 13.86 | 6.71 | 12.00 | 4.89 | 0.50 | 0.07 | 0.42 | 3.35 | 0.36 | 0.06 | 0.84 | 0.00 | 1.00 | 1.50 | 1.33 | 1.43 | 1.01 | 1.50 | 1.20 | 1.50 | 1.68 |
| 102.014 | 30.533 | 0.86 | 4.81 | 3.21 | 0.93 | 12.00 | 10.00 | 14.81 | 6.19 | 11.78 | 5.00 | 0.48 | 0.08 | 0.41 | 3.15 | 0.36 | 0.04 | 0.82 | 0.00 | 1.00 | 1.47 | 1.31 | 1.40 | 1.00 | 1.48 | 1.21 | 1.48 | 1.66 |
| 101.954 | 30.418 | 0.84 | 5.38 | 4.11 | 0.88 | 11.37 | 9.08 | 12.72 | 6.45 | 11.56 | 4.35 | 0.45 | 0.10 | 0.45 | 3.10 | 0.36 | 0.06 | 0.89 | 0.00 | 1.04 | 1.50 | 1.38 | 1.46 | 1.05 | 1.50 | 1.20 | 1.43 | 1.72 |
| 102.048 | 30.471 | 0.75 | 5.19 | 4.45 | 0.78 | 10.88 | 8.61 | 11.39 | 5.94 | 11.33 | 3.80 | 0.43 | 0.55 | 0.42 | 2.86 | 0.40 | 0.03 | 0.94 | 0.00 | 1.04 | 1.50 | 1.15 | 1.28 | 0.94 | 1.43 | 1.24 | 1.02 | 1.94 |
| 101.995 | 30.609 | 0.80 | 5.02 | 4.35 | 0.85 | 10.48 | 8.83 | 9.49 | 5.68 | 11.11 | 3.91 | 0.40 | 0.51 | 0.43 | 2.94 | 0.40 | 0.01 | 0.99 | 0.00 | 1.08 | 1.40 | 1.27 | 1.32 | 0.94 | 1.43 | 1.22 | 1.06 | 1.98 |
| 101.881 | 31.091 | 0.75 | 6.88 | 4.22 | 0.75 | 10.82 | 6.87 | 9.08 | 6.71 | 10.89 | 3.26 | 0.45 | 0.62 | 0.44 | 2.70 | 0.37 | 0.01 | 1.06 | 0.00 | 1.09 | 1.39 | 1.21 | 1.15 | 0.89 | 1.49 | 1.24 | 0.92 | 2.00 |
| 102.154 | 30.377 | 0.76 | 3.76 | 5.00 | 0.71 | 11.28 | 9.53 | 7.22 | 8.00 | 9.56 | 2.93 | 0.40 | 0.40 | 0.44 | 3.22 | 0.41 | 0.02 | 0.92 | 0.00 | 1.09 | 1.43 | 1.30 | 1.32 | 0.92 | 1.40 | 1.23 | 1.24 | 1.87 |
| 101.365 | 32.601 | 0.77 | 3.24 | 3.70 | 0.90 | 9.91 | 9.77 | 10.63 | 5.16 | 10.44 | 4.13 | 0.35 | 0.34 | 0.47 | 3.59 | 0.43 | 0.01 | 1.04 | 0.00 | 1.08 | 1.40 | 1.21 | 1.21 | 0.91 | 1.35 | 1.24 | 1.19 | 1.86 |
| 101.819 | 31.012 | 0.74 | 4.63 | 3.87 | 0.83 | 10.28 | 8.33 | 10.06 | 5.42 | 10.00 | 4.24 | 0.38 | 0.31 | 0.46 | 3.02 | 0.43 | 0.01 | 0.99 | 0.00 | 1.13 | 1.40 | 1.27 | 1.24 | 0.98 | 1.35 | 1.22 | 1.16 | 1.84 |
| 101.461 | 32.585 | 0.76 | 4.98 | 3.59 | 0.95 | 9.21 | 9.39 | 8.35 | 4.90 | 10.89 | 4.57 | 0.33 | 0.38 | 0.49 | 3.43 | 0.47 | 0.04 | 0.84 | 0.00 | 1.13 | 1.40 | 1.44 | 1.35 | 0.98 | 1.20 | 1.22 | 1.26 | 1.90 |
| 101.928 | 31.418 | 0.76 | 4.76 | 3.46 | 0.99 | 8.84 | 9.30 | 7.78 | 4.65 | 10.67 | 4.67 | 0.30 | 0.27 | 0.50 | 4.00 | 0.50 | 0.04 | 0.94 | 0.00 | 1.21 | 1.40 | 1.50 | 1.50 | 1.01 | 1.13 | 1.22 | 1.30 | 1.92 |
| 101.977 | 31.261 | 0.70 | 5.07 | 3.33 | 1.00 | 8.38 | 8.96 | 5.51 | 4.39 | 10.44 | 4.78 | 0.27 | 0.49 | 0.48 | 3.82 | 0.49 | 0.03 | 0.87 | 0.00 | 1.18 | 1.43 | 1.38 | 1.42 | 0.89 | 1.24 | 1.25 | 1.33 | 1.96 |
| 101.879 | 31.846 | 0.63 | 4.58 | 2.24 | 0.40 | 9.51 | 7.39 | 11.96 | 4.13 | 9.78 | 0.33 | 0.38 | 1.40 | 0.01 | 1.80 | 0.12 | 0.25 | 0.20 | 0.00 | 1.21 | 0.62 | 0.92 | 0.95 | 1.33 | 0.98 | 1.41 | 1.19 | 0.15 |
| 102.978 | 31.766 | 0.58 | 3.39 | 1.70 | 0.40 | 9.39 | 5.87 | 15.00 | 2.58 | 8.00 | 0.43 | 0.14 | 1.05 | 0.06 | 1.47 | 0.07 | 0.20 | 0.18 | 0.00 | 1.45 | 0.59 | 0.85 | 0.70 | 1.48 | 1.01 | 1.46 | 1.46 | 0.02 |
| 103.103 | 32.265 | 0.58 | 3.66 | 1.59 | 0.33 | 9.21 | 6.51 | 12.15 | 3.35 | 8.67 | 0.33 | 0.18 | 1.16 | 0.07 | 1.39 | 0.12 | 0.18 | 0.20 | 0.00 | 1.42 | 0.62 | 0.92 | 0.73 | 1.43 | 1.13 | 1.48 | 1.33 | 0.05 |
| 100.888 | 31.568 | 0.59 | 4.07 | 2.82 | 0.73 | 6.99 | 9.98 | 10.63 | 2.84 | 7.11 | 0.22 | 0.20 | 1.32 | 0.04 | 1.86 | 0.09 | 0.24 | 0.17 | 0.00 | 1.32 | 0.57 | 0.88 | 0.81 | 1.40 | 1.00 | 1.46 | 1.22 | 0.13 |
| 103.676 | 32.119 | 0.55 | 7.00 | 1.47 | 0.11 | 7.03 | 7.92 | 4.75 | 5.94 | 9.56 | 1.09 | 0.27 | 1.46 | 0.01 | 2.05 | 0.14 | 0.24 | 0.10 | 0.00 | 1.34 | 0.54 | 0.78 | 0.87 | 1.30 | 1.12 | 1.43 | 1.10 | 0.24 |
| 103.158 | 31.507 | 0.52 | 3.86 | 2.33 | 0.47 | 7.70 | 8.33 | 10.06 | 3.87 | 7.78 | 0.65 | 0.25 | 1.23 | 0.03 | 1.80 | 0.12 | 0.22 | 0.15 | 0.00 | 1.29 | 0.72 | 0.75 | 0.84 | 1.36 | 1.05 | 1.44 | 1.26 | 0.09 |
| 103.648 | 31.794 | 0.53 | 3.90 | 1.85 | 0.41 | 7.33 | 7.71 | 8.73 | 3.61 | 8.22 | 0.54 | 0.30 | 1.30 | 0.05 | 1.96 | 0.08 | 0.19 | 0.15 | 0.00 | 1.25 | 0.62 | 0.81 | 0.80 | 1.26 | 0.90 | 1.44 | 1.16 | 0.07 |
| 102.057 | 30.085 | 0.58 | 4.42 | 1.22 | 0.24 | 6.76 | 7.37 | 7.59 | 4.39 | 9.11 | 0.87 | 0.33 | 1.40 | 0.01 | 1.63 | 0.05 | 0.21 | 0.05 | 0.00 | 1.38 | 0.52 | 0.69 | 0.77 | 1.33 | 0.98 | 1.41 | 1.02 | 0.19 |

| | | | | | | | | | | | | | | | | | | | | | | | | | | | | |
|---|---|---|---|---|---|---|---|---|---|---|---|---|---|---|---|---|---|---|---|---|---|---|---|---|---|---|---|---|
| 102.182 | 30.171 | 0.54 | 3.11 | 0.69 | 0.40 | 8.84 | 6.00 | 9.68 | 3.10 | 7.56 | 0.65 | 0.13 | 1.09 | 0.08 | 1.22 | 0.15 | 0.16 | 0.25 | 0.00 | 1.50 | 0.72 | 1.10 | 0.66 | 1.50 | 1.20 | 1.50 | 1.40 | 0.03 |
| 103.201 | 32.041 | 0.58 | 4.19 | 1.12 | 0.31 | 6.62 | 7.18 | 5.70 | 4.90 | 8.89 | 0.76 | 0.35 | 1.43 | 0.03 | 1.55 | 0.05 | 0.22 | 0.04 | 0.00 | 1.33 | 0.52 | 0.75 | 0.84 | 1.22 | 1.05 | 1.43 | 0.99 | 0.17 |
| 101.187 | 30.998 | 0.42 | 3.25 | 0.21 | 0.61 | 6.61 | 4.55 | 1.52 | 5.94 | 6.67 | 0.54 | 0.20 | 1.46 | 0.05 | 2.07 | 0.07 | 0.27 | 0.24 | 0.00 | 1.18 | 0.59 | 1.02 | 0.86 | 1.40 | 0.86 | 1.50 | 1.11 | 0.13 |
| 101.981 | 32.066 | 0.43 | 2.96 | 0.26 | 0.57 | 6.22 | 5.37 | 3.23 | 5.16 | 6.00 | 0.43 | 0.23 | 1.26 | 0.04 | 1.71 | 0.01 | 0.24 | 0.10 | 0.00 | 1.13 | 0.72 | 0.98 | 0.91 | 1.29 | 0.83 | 1.43 | 1.06 | 0.11 |
| 102.056 | 29.949 | 0.33 | 1.96 | 0.77 | 0.03 | 2.82 | 2.01 | 13.29 | 2.32 | 5.11 | 0.11 | 0.02 | 1.04 | 0.19 | 0.64 | 0.01 | 0.45 | 1.80 | 0.00 | 0.03 | 0.03 | 0.12 | 0.06 | 0.02 | 0.25 | 0.01 | 0.20 | 0.72 |
| 103.707 | 31.876 | 0.26 | 3.35 | 0.66 | 0.21 | 2.32 | 1.85 | 12.34 | 1.81 | 4.44 | 0.09 | 0.05 | 1.09 | 0.18 | 0.57 | 0.05 | 0.45 | 1.74 | 0.00 | 0.02 | 0.13 | 0.06 | 0.04 | 0.02 | 0.30 | 0.00 | 0.17 | 0.75 |
| 101.833 | 30.777 | 0.26 | 1.57 | 0.71 | 0.01 | 2.83 | 1.93 | 12.72 | 2.06 | 4.89 | 0.09 | 0.01 | 1.02 | 0.17 | 0.73 | 0.01 | 0.43 | 1.69 | 0.00 | 0.02 | 0.13 | 0.05 | 0.03 | 0.02 | 0.38 | 0.00 | 0.20 | 0.79 |
| 102.791 | 31.411 | 0.34 | 5.57 | 0.56 | 0.08 | 1.42 | 1.36 | 9.49 | 1.03 | 3.33 | 0.22 | 0.08 | 1.06 | 0.20 | 0.49 | 0.08 | 0.49 | 1.94 | 0.00 | 0.04 | 0.03 | 0.23 | 0.26 | 0.10 | 0.15 | 0.02 | 0.31 | 0.57 |
| 103.773 | 32.206 | 0.23 | 3.54 | 0.63 | 0.04 | 1.80 | 1.65 | 11.96 | 0.26 | 2.44 | 0.11 | 0.15 | 0.92 | 0.21 | 0.41 | 0.05 | 0.46 | 1.79 | 0.00 | 0.02 | 0.13 | 0.12 | 0.11 | 0.03 | 0.23 | 0.02 | 0.24 | 0.67 |
| 103.597 | 31.892 | 0.26 | 3.61 | 0.81 | 0.05 | 0.99 | 1.75 | 11.39 | 0.21 | 1.78 | 0.11 | 0.10 | 0.81 | 0.20 | 0.42 | 0.10 | 0.50 | 2.00 | 0.00 | 0.04 | 0.01 | 0.25 | 0.25 | 0.12 | 0.04 | 0.01 | 0.40 | 0.49 |
| 101.945 | 30.812 | 0.33 | 3.62 | 0.61 | 0.08 | 1.38 | 1.29 | 8.80 | 1.60 | 2.62 | 0.18 | 0.08 | 1.11 | 0.21 | 0.38 | 0.08 | 0.47 | 1.91 | 0.00 | 0.05 | 0.01 | 0.17 | 0.16 | 0.07 | 0.15 | 0.01 | 0.22 | 0.61 |
| 102.891 | 31.339 | 0.29 | 3.45 | 0.59 | 0.07 | 1.55 | 1.23 | 10.44 | 0.52 | 2.22 | 0.22 | 0.13 | 0.89 | 0.21 | 0.33 | 0.08 | 0.47 | 1.89 | 0.00 | 0.04 | 0.03 | 0.17 | 0.15 | 0.03 | 0.23 | 0.02 | 0.27 | 0.65 |
| 103.514 | 31.665 | 0.28 | 3.51 | 0.51 | 0.09 | 1.03 | 0.62 | 6.65 | 0.77 | 3.78 | 0.33 | 0.10 | 0.99 | 0.22 | 0.24 | 0.05 | 0.49 | 1.84 | 0.00 | 0.02 | 0.13 | 0.23 | 0.22 | 0.07 | 0.15 | 0.04 | 0.34 | 0.55 |
| 101.888 | 31.317 | 0.17 | 3.95 | 0.26 | 0.15 | 0.33 | 0.12 | 2.66 | 2.06 | 0.89 | 0.43 | 0.14 | 1.47 | 0.24 | 0.09 | 0.14 | 0.46 | 1.92 | 0.00 | 0.09 | 0.01 | 0.13 | 0.18 | 0.07 | 0.16 | 0.01 | 0.06 | 0.62 |
| 101.001 | 31.517 | 0.16 | 3.68 | 0.36 | 0.11 | 0.52 | 0.21 | 2.85 | 1.29 | 0.44 | 0.54 | 0.18 | 1.33 | 0.25 | 0.16 | 0.12 | 0.45 | 1.84 | 0.00 | 0.08 | 0.13 | 0.17 | 0.22 | 0.07 | 0.08 | 0.02 | 0.14 | 0.59 |
| 100.915 | 31.469 | 0.15 | 3.84 | 0.28 | 0.13 | 0.26 | 0.09 | 1.90 | 1.55 | 0.36 | 0.65 | 0.20 | 1.50 | 0.26 | 0.07 | 0.15 | 0.43 | 1.89 | 0.00 | 0.08 | 0.13 | 0.29 | 0.29 | 0.14 | 0.03 | 0.00 | 0.05 | 0.63 |

**Table S4**. Types of *M. esculenta* nutritional components and their weighting ratios

| Index | Nutritional Component | Weight (%) | Index | Nutritional Component | Weight (%) |
|---|---|---|---|---|---|
| 1 | Ash | 1 | 14 | Tyrosine | 1.5 |
| 2 | Protein | 12 | 15 | Arginine | 2 |
| 3 | Dietary Fiber | 10 | 16 | Glutamate | 4 |
| 4 | Fat | 7 | 17 | Aspartic Acid | 0.5 |
| 5 | Crude Fiber | 5 | 18 | Serine | 0.5 |
| 6 | Starch | 15 | 19 | Glycine | 0.5 |
| 7 | Total Flavonoids | 12 | 20 | Alanine | 0.5 |
| 8 | Total Saponins | 8 | 21 | Proline | 2 |
| 9 | Tannins | 5 | 22 | Cysteine | 1.5 |
| 10 | Threonine | 1.5 | 23 | Leucine | 1.5 |
| 11 | Valine | 1.5 | 24 | Phenylalanine | 1.5 |
| 12 | Methionine | 1.5 | 25 | Lysine | 1.5 |
| 13 | Isoleucine | 1.5 | 26 | Histidine | 1.5 |
